# Intramuscular cavernous malformation in the temporalis muscle: Diagnosis and treatment of a rare tumor in a rare site

**DOI:** 10.1002/ccr3.8267

**Published:** 2023-11-27

**Authors:** Mohammad Jafarian, Mohammad Amir Alizadeh Tabrizi, Fatemeh Mashhadi Abbas, Zahra Sadat Torabi

**Affiliations:** ^1^ Department of Oral and Maxillofacial Surgery Taleghani University Hospital, Shahid Beheshti University of Medical Sciences Tehran Iran; ^2^ Department of Oral and Maxillofacial Surgery, School of Dentistry Zahedan University of Medical Sciences Zahedan Iran; ^3^ Department of Oral and Maxillofacial Pathology Shahid Beheshti University of Medical Sciences Tehran Iran

**Keywords:** cavernous hemangioma, intramuscular, temporalis muscle

## Abstract

In this case report we present a very rare case of intramuscular cavernous hemangioma in the temporalis muscle which was successfully managed with surgical excision with no evidence of recurrence in follow‐up.

## Graphical abstract

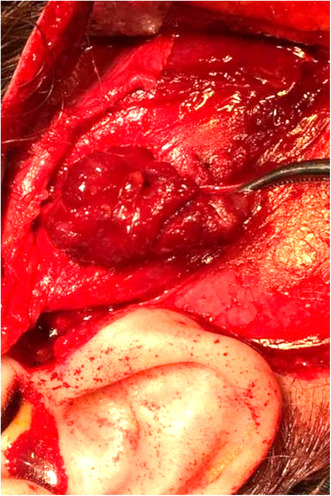

## 1 INTRODUCTION

Vascular malformations are a diverse group of pathologic lesions. The most common types of vascular malformations are capillary telangiectasias, cavernous malformations, venous malformations, and arteriovenous malformations.^1^ Hemangiomas are benign neoplasms with vascular origin typically located in skin or subcutaneous tissues. They represent 7% of all nonmalignant soft tissue neoplasms. Less than 1% of hemangiomas arise from the muscular structures. The most commonly involved areas are large skeletal muscles of the lower extremities and only 10% of intramuscular hemangiomas are located in the head and neck area.^2^ Moreover, the most common site of intramuscular hemangioma in the head and neck is the masseter muscle, and temporalis muscle involvement is rare.^3^ Thus, presentation of this pathologic lesion in the temporal area might be largely confusing in the clinical practice. Here, successful diagnosis and management of a case of an intramuscular cavernous hemangioma (ICH) of the temporalis muscle is reported.

## 2 CASE HISTORY

**FIGURE 1:**
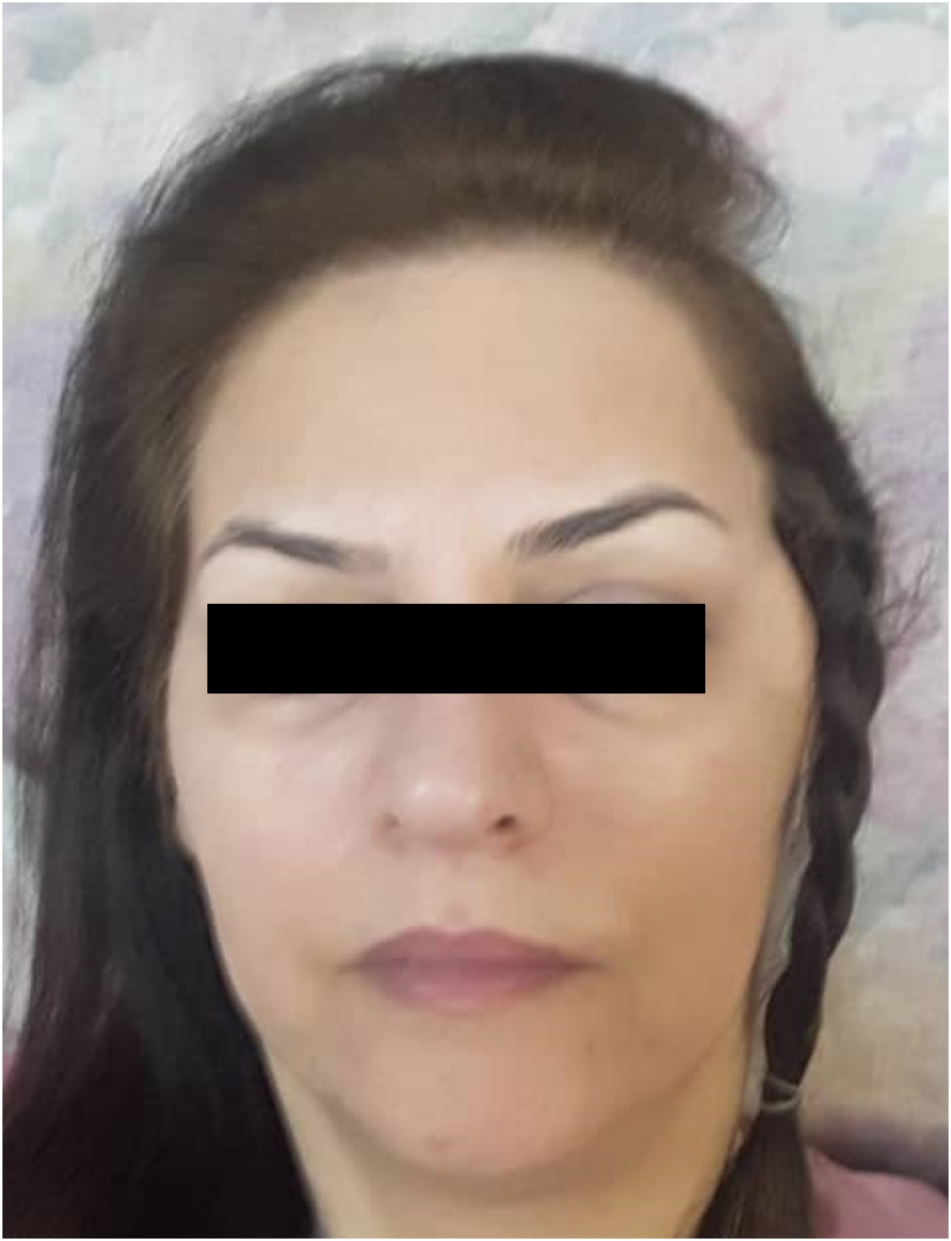
Pre‐operative image of the patient showing a bulk in her left temporalis muscle.

A 51‐year‐old female was admitted to the oral and maxillofacial surgery department of a tertiary university hospital in August 2019, complaining about the presence of a mass in her left temporal area (Figure 1). The patient had noticed the lesion since 10 years ago and reported a slight gradual increase in its size, but she preferred not to seek any medical consultation because the mass was painless. The patient reported a mild unpleasant feeling of pressure while talking or chewing food. She presented no history of trauma to the region. The patient only reported a history of pregnancy followed by a cesarean section 27 years ago and the past medical history, familial history, and drug history of the patient were otherwise, unremarkable.

Clinical examination revealed a rubbery, compressible, painless, immobile, dome‐shaped mass of 2.5 cm in diameter, above the left zygomatic arch. No pulsation or bruit was detected and the overlying skin was normal with no signs of erythema or warmth. The mass became slightly more apparent when the patient clenched her jaw but there was no clear change in the size of the lesion with Valsalva maneuver. Mandibular movements were within normal range and not restricted. Neurologic examination of cranial nerves was also normal. No adenopathy was found in the face and neck area examination.

**FIGURE 2:**
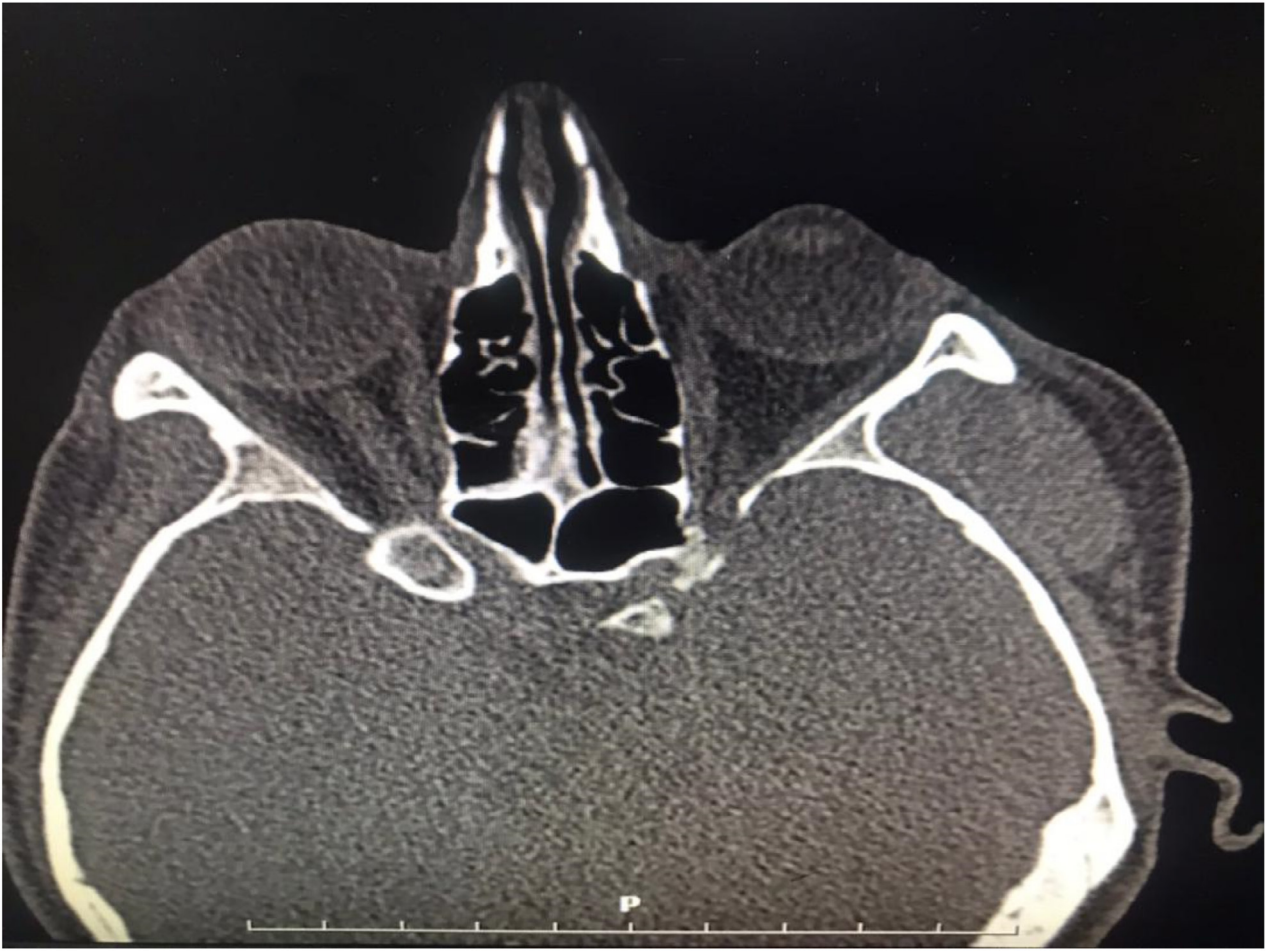
Computed tomography scan showing a mass lesion in the left temporalis muscle.

**FIGURE 3:**
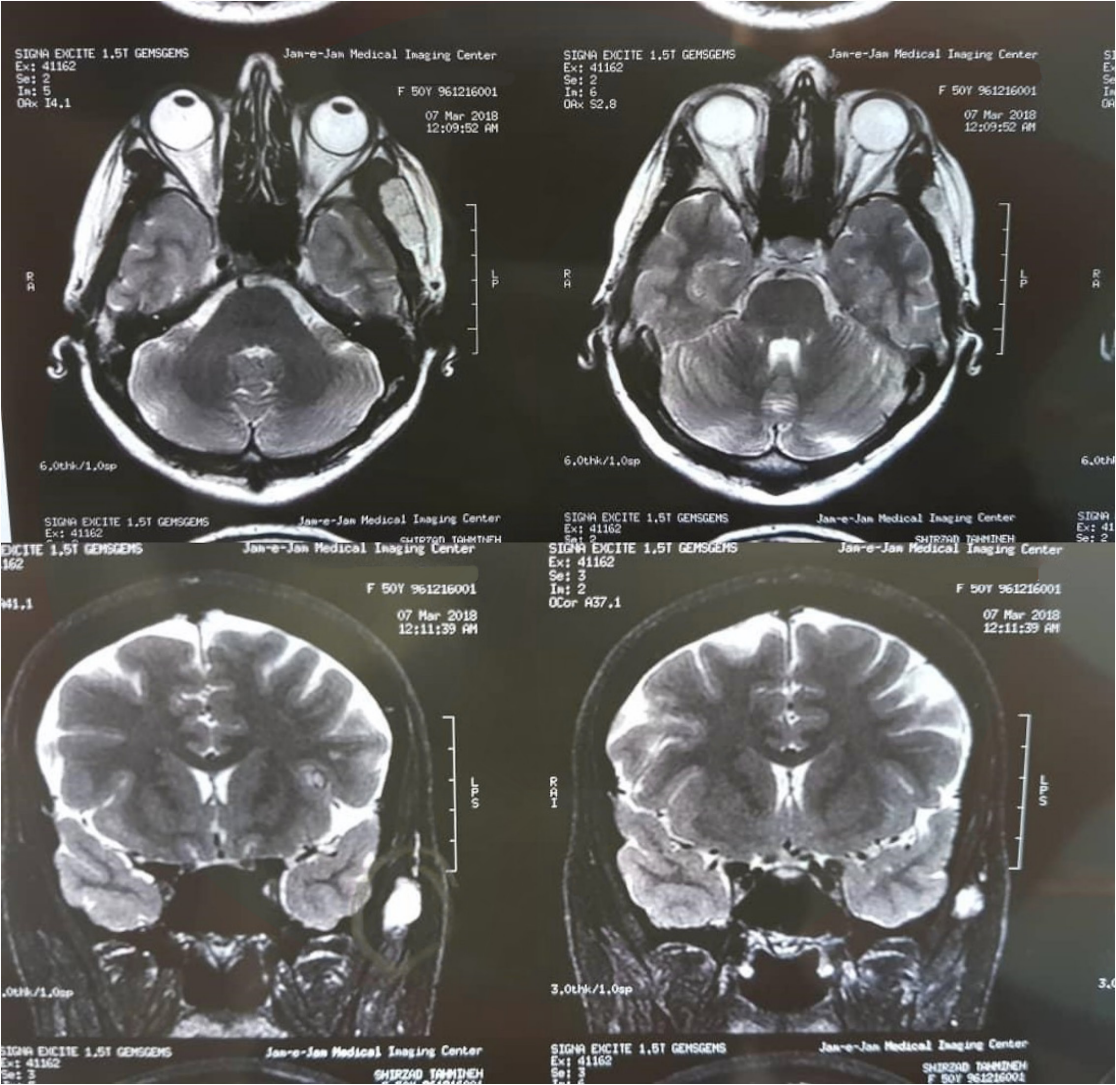
Axial (above) and coronal (below) T2 sequence magnetic resonance imaging showing a high‐intensity lesion within the left temporalis muscle.

For radiologic examination, computed tomography(CT) scan of the lesion showed a well‐defined soft tissue mass in the temporal region with no bony erosion or invasion (Figure 2). Subsequently, a magnetic resonance image(MRI) was acquired which displayed a 24 × 12 mm high‐intensity signal lesion on T1 and T2‐weighted images, with lobulated borders and linear low‐intensity internal structures in the anterior inferior aspect of the temporalis muscle (Figure 3). These findings were suggestive of an angiomatous lesion. The patient declined further angiographic examinations due to financial reasons.

**FIGURE 4:**
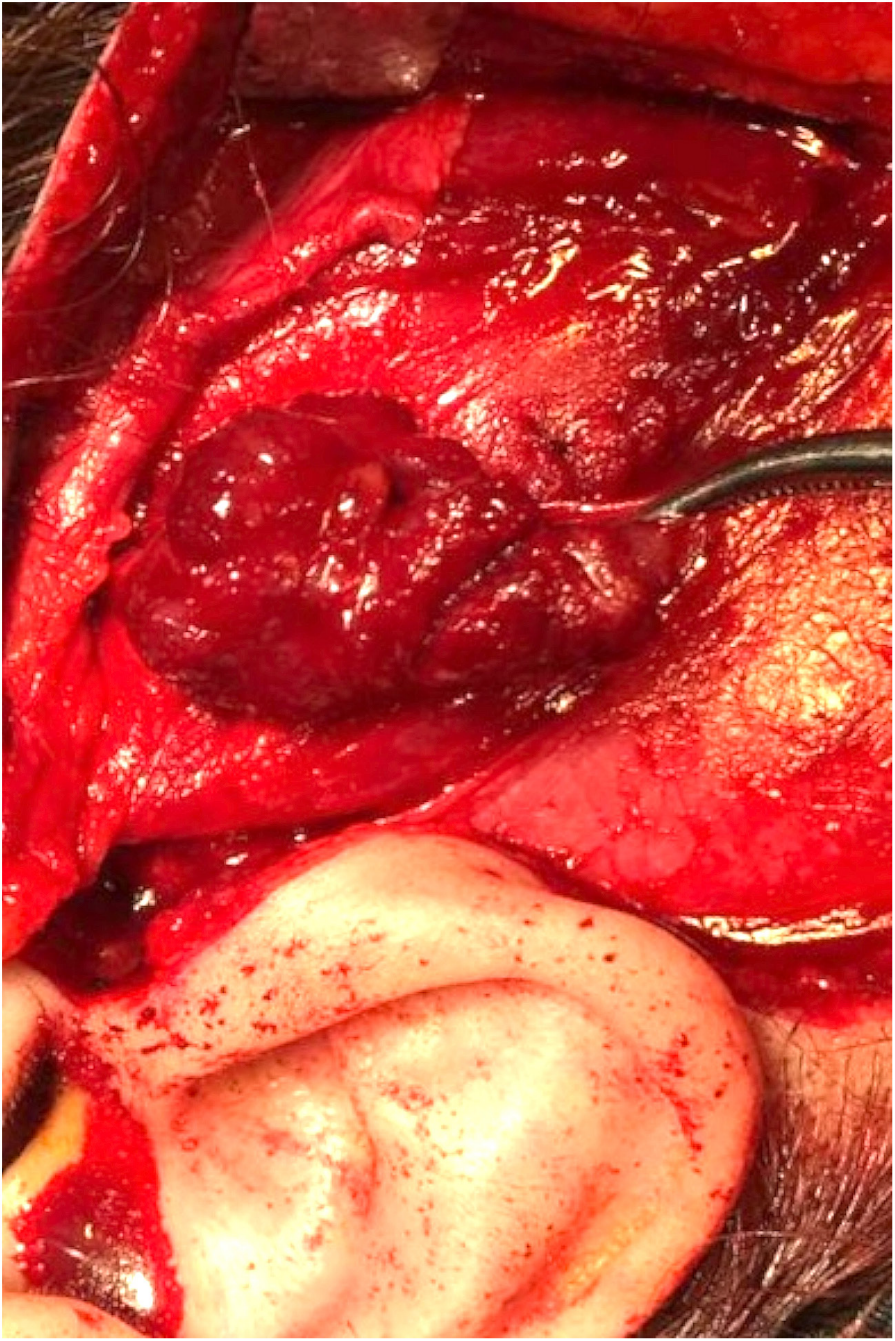
Intra‐operative image showing the lesion in the caudal aspect of the temporalis muscle.

After acquiring informed consent, the patient underwent full surgical excision of the lesion under general anesthesia. Al‐Kayat Bramley incision^4^ was used for comprehensive access to the lesion and avoiding visible scar while preserving temporal branches of the facial nerve (Figures 4,, 5). Intraoperative examination revealed a lobulated soft to elastic blueish‐red mass in the temporalis muscle above the zygomatic arch without any invasion to the surrounding structures. Enlarged vascular structures were revealed in the inferior aspect of the lesion after surgical dissection which were ligated. The postoperative clinical course was eventless and there was no temporary or permanent weakness of the facial nerve.

**FIGURE 5:**
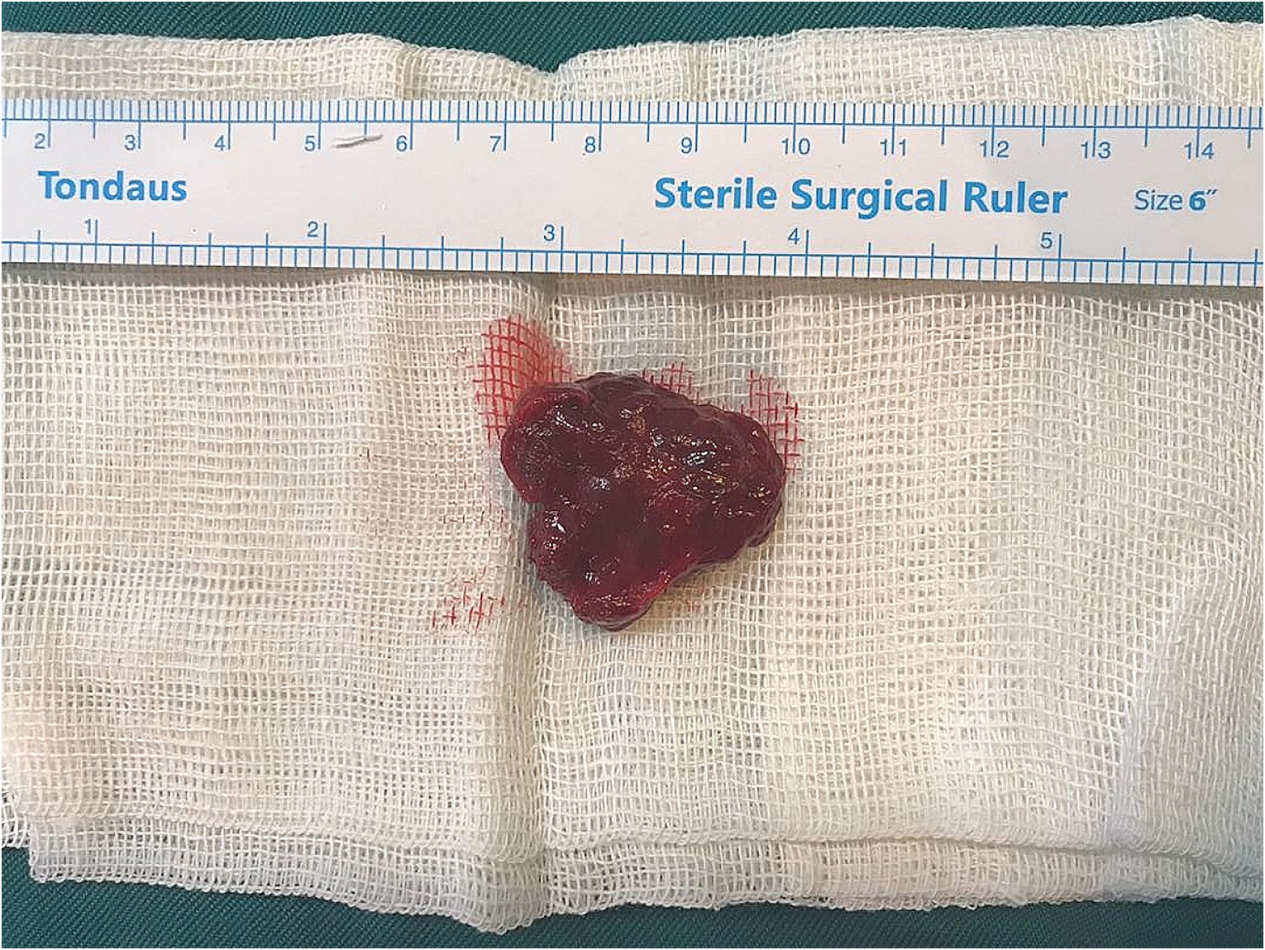
Clinical image of the gross lesion after excision.

**FIGURE 6:**
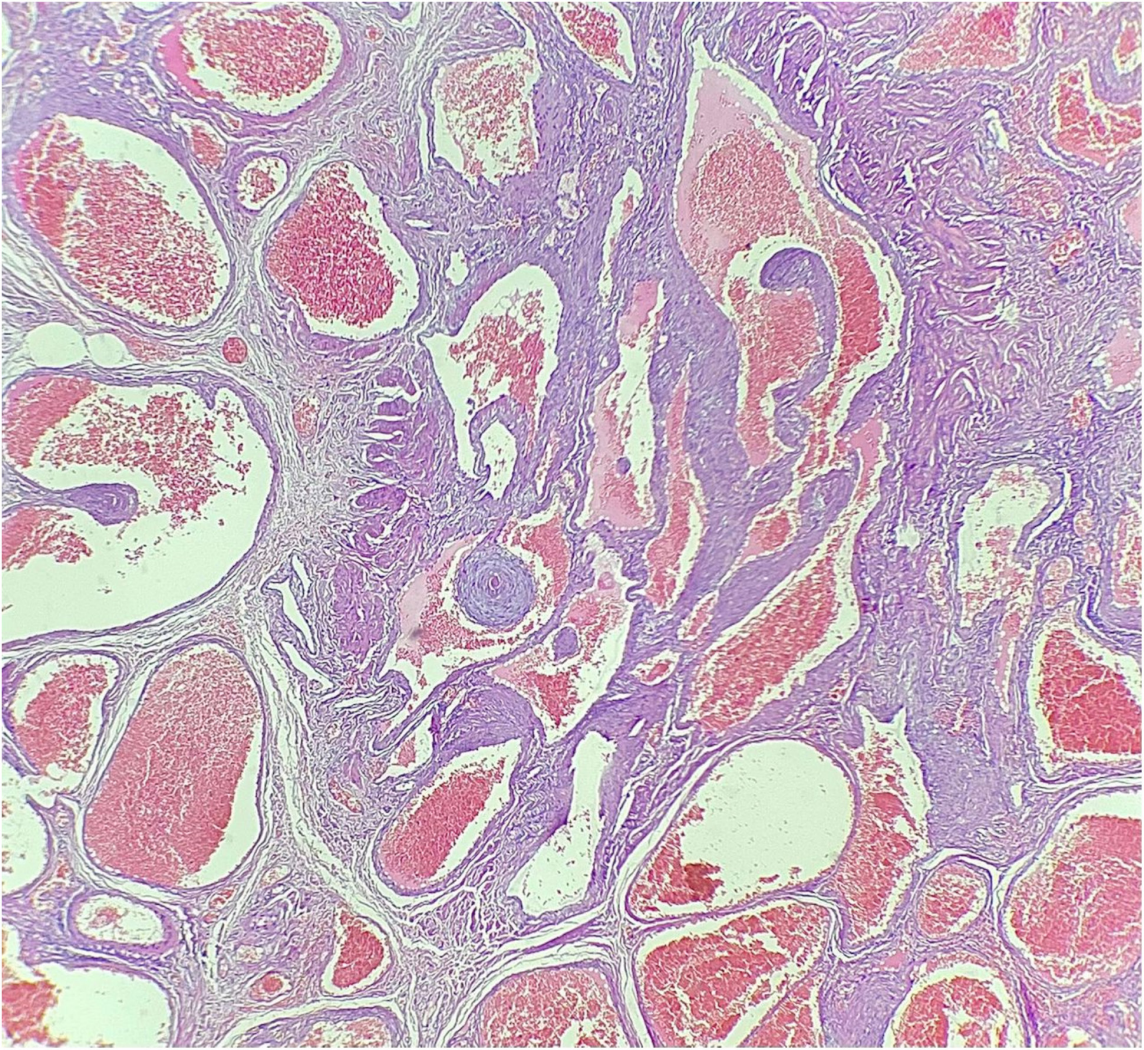
Microscopic image showing multiple large vessels in a fibrotic stroma.

Gross pathologic examination showed one piece of lobular encapsulated grayish‐brown soft to elastic tissue measuring 3 × 2.5 × 1.5 cm with variable‐sized cystic spaces filled by fragile brown material. Histologic examination of the lesion revealed a vascular lesion consisting of numerous large endothelium‐lined vessels with intraluminal papillary projections, sinusoidal, and anastomosing vessels in a fibrotic stroma. Intravascular thrombosis, adipose tissue, and patchy inflammatory cell infiltration were also seen (Figure 6). There was no sign of malignancy. The final diagnosis was intra‐muscular cavernous malformation.

The patient was followed up periodically for up to 2 years with no sign of recurrence and no limitation in mandibular movements.

## 3 DISCUSSION

There are very few reported cases of intramuscular hemangioma in the temporalis muscle with the first cases dating back to the 1950s.^5^

Unlike the common subcutaneous types of hemangioma, intramuscular hemangioma is not present at birth, is usually detected in the second or third decades of life, tends to grow slowly and progressively, and does not show spontaneous regression. Although the masseter muscle is the most common site of intramuscular hemangioma of the head region,^6^ this lesion has been reported in other facial muscles such as the zygomatic,^7^ buccinator,^8^ extraocular,^9^ and temporalis muscle.^10^

Hemangiomas are divided into five common categories based on their predominant histologic vasculature: capillary (small vessel), cavernous (large vessel), arteriovenous, venous, and mixed.^11^ Cavernous hemangiomas, also called cavernous malformations, are clusters of abnormally dilated and cyst‐like capillary structures with flattened endothelium, whereas capillary hemangiomas consist of highly cellular small‐sized vessels. Cavernous hemangiomas tend to grow slower so the patient usually notes a longer history of the lesion.^12^

In a literature review by Alqahtani et al.^13^ on 33 temporalis muscle hemangioma cases reported until 2022, 18/33 patients were males indicating an almost even gender distribution. Besides trauma, hormonal factors are also cited as potential etiologic entities, especially with existing evidence indicating stable or regressing lesions in postmenopausal female patients.^14^ Despite this, there is still controversy regarding the role of estrogen and progesterone receptors in the development of cavernous hemangiomas.^15^, ^16^

These lesions can easily be diagnosed by MRI with typical hypersignal images on T2 and hypo‐to‐iso signal images on T1 sequences.^17^ Any form of biopsy could potentially lead to substantial bleeding as well as yielding inadequate specimen and therefore is not recommended.

Although many consider surgery as the best treatment option for intramuscular hemangiomas of the temporalis muscle,^18^, ^19^ some recommend it, only if the patient has cosmetic complaints or the lesion is suspected to be capillary hemangioma which is more likely to be infiltrative and recurrent.^20^ On clinical grounds, this can be suspected by a lack of Valsalva dependence of lesion dimension changes or radiologic signs. During surgical excision, care should be given to spare facial nerve branches. Our patient had neither Valsalva dependence nor was she cosmetically satisfied with her appearance and therefore we decided to proceed with complete surgical excision. In accord with the successful surgical management of these lesions reported by previous studies, our patient was cured as well with no evidence of recurrence or surgery‐associated adverse events with 2 years of follow‐up. Other treatment options for intramuscular hemangiomas include embolization,^21^ corticosteroids,^22^ beta‐blockers,^23^ and radiation therapy^24^ although with variable responses and seemingly inferior to definitive surgery as none of these methods removes the lesion completely.

Following the experience with the present case and a few others reported in the literature, it is recommended to consider intramuscular hemangioma in the differential diagnosis of isolated pathologic lesions of facial muscles when radiologic and clinical symptoms are in accord with a benign neoplasm of vascular origin. Surgical treatment is generally successful with very low rates of recurrence.

## 4 CONCLUSION

In this article, we reported a rare case of intramuscular cavernous malformation in the temporalis muscle; an unusual site with very few cases reported in the literature. The patient was successfully treated with complete surgical excision of the lesion with preservation of the facial nerve branches. There is no evidence of disease recurrence or treatment‐related adverse events with 2 years of follow

## AUTHOR CONTRIBUTIONS

**Mohammad Jafarian:** Conceptualization; data curation; project administration; supervision; validation; writing – original draft; writing – review and editing. **Mohammad Amir Alizadeh Tabrizi:** Conceptualization; data curation; project administration; resources; writing – original draft; writing – review and editing. **Fatemeh Mashhadi Abbas:** Data curation; resources; supervision; writing – original draft; writing – review and editing. **Zahra Sadat Torabi:** Conceptualization; data curation; investigation; project administration; resources; writing – original draft; writing – review and editing.

## FUNDING INFORMATION

None.

## CONFLICT OF INTEREST STATEMENT

None.

## ETHICS STATEMENT

Our study was performed in line with the principles of the Declaration of Helsinki. The subject signed informed consent and patient anonymity was preserved.

## CONSENT

Written informed consent was obtained from the patient to publish this report in accordance with the journal's patient consent policy.

## Data Availability

Research data are stored in an institutional repository and will be shared upon request to the corresponding author.
